# Identification of FOXM1 and CXCR4 as key genes in breast cancer prevention and prognosis after intermittent energy restriction through bioinformatics and functional analyses

**DOI:** 10.1080/21623945.2022.2069311

**Published:** 2022-05-19

**Authors:** Lusha Li, Liangli Chen, Li Yu, Junlu Zhang, Liying Chen

**Affiliations:** a Department of General Practice, Sir Run Run Shaw Hospital, School of Medicine, Zhejiang University, Hangzhou Zhejiang, China; b Zhejiang University, Hangzhou Zhejiang, China

**Keywords:** Intermittent energy restriction, obesity, breast cancer, differentially expressed genes, enrichment analysis, protein–protein interaction network, hub genes, bioinformatics

## Abstract

We explored potential biomarkers and molecular mechanisms regarding breast cancer (BC) risk reduction after intermittent energy restriction (IER) and further explored the association between IER and BC prognosis. We identified differentially expressed genes (DEGs) in breast tissues before and after IER by analyzing the expression profile from GEO. Then, enrichment analysis was used to identify important pathways of DEGs and hub genes were selected from PPI network. After that, GEPIA, ROC, and KM plotter were used to explore the preventive and prognostic value of hub genes. It was found that FOXM1 and CXCR4 were highly expressed in BC tissues and associated with the worse prognosis. FOXM1 and CXCR4 were down-regulated after IER , which meant that FOXM1 and CXCR4 might be the most important key genes for reducing the risk and improving prognosis of BC after IER . ROC curve indicated that FOXM1 and CXCR4 also had the predictive value for BC. Our study contributed to a better understanding of the specific mechanisms in protective effects of IER on BC and provided a new approach to improve the prognosis of BC, which might provide partial guidance for the subsequent development of more effective treatments and prevention strategies.

## 1. Introduction

As the most common cancer among women, breast cancer (BC) poses a huge threat to women’s health and is the leading cause of cancer-related deaths worldwide [1]. Obesity is a state of over-nourishment, which chronically activates the cell growth factor signalling pathways and increases the risk of neoplastic transformation [2]. Several cohort studies have shown that both weight gain and obesity in mid-life increase the risk of postmenopausal BC [3–5]. Women who gained 20 kg during mid-life double their risk of developing BC after menopause compared to women who kept their weight [6]. The increased risk of BC in overweight or obese women after menopause is thought to be a direct result of higher energy supply and positive energy balance [7,8]. Energy restriction (ER) significantly reduces the occurrence of BC in laboratory rodents regardless of the type of nutritional restriction [9]. There have been found a variety of cellular changes in animal studies, such as alteration of growth factors, signalling and metabolic pathways and reduction of cell proliferation [10,11]. Observational studies show that weight loss is beneficial for reducing the risk of postmenopausal BC in both premenopausal and postmenopausal women [5,12]. In most studies of ER, the restriction regimen involved is daily continuous energy restriction (CER; reducing energy supply by about 500 or 750 kcal per day, or reducing 30% energy supply of baseline requirement). However, the well-known problem is that achieving and maintaining weight loss through CER is difficult to persist in because of subjects’ poor compliance and a vast majority of people will regain weight within a year [13,14]. Intermittent energy restriction (IER; delaying energy supply for the next meal) has become popular in the years because of its benefits in achieving weight loss and some metabolism-related health improvements, and has been suggested as a possible alternative approach of CER. However, clinical studies of IER intervention in overweight and obesity women are limited. The underlying mechanism by which IER intervention reduces postmenopausal BC risk is unclear. Further evidences show that obesity in women is related to the worse prognosis of BC [15,16]. Although literatures searching reveals that there is no current study on the association between IER and prognosis of BC, Dewhirst et al [17]. and Zhang et al [18]. have found that as tumours continue to growing, cancer cells compete fiercely for increased metabolic demands in a microenvironment with limited nutrient and energy supplies. We speculated that IER would improve BC outcome by inhibiting metabolic process and the potential targets for prevention of BC after IER intervention may also be the potential targets for improving prognosis of BC.

With the fast development and continuous progress of high-throughput system (HTS) technology, integrated bioinformatics analysis is becoming more and more popular as a new method to explore the mechanism of IER intervention. Harvie MN et al., generated the GSE66161 [19] microarray dataset, which compared gene expression on the breast tissue before and after using IER intervention (2 days of 65% energy restriction) for one menstrual cycle in 20 overweight or obese women. In our study, we aimed to further investigate how the potential key genes and pathways changing in breast tissue after IER intervention through bioinformatic analysis in order to provide potential targets for the prevention and prognosis of BC. By analysing GSE66161, differentially expressed genes (DEGs) were identified and used for subsequent functions and pathways enrichment. We then constructed the protein-protein interaction (PPI) network and identified the hub genes from it through the MCODE plug-in. Attie Lab Diabetes and GEPIA were used to investigate expression of hub genes in obesity and whether the expression levels of hub genes in normal and BC tissues were different, respectively. After that, we further evaluated the area under curve (AUC) of receiver operating characteristic (ROC) in GSE70947 to explore the predictive value of hub genes for postmenopausal BC risk [20]. The expression profiles of GSE70947 consisted of 210 samples of BC tissues from postmenopausal women and matched the nearby normal breast tissues. Finally, Kaplan–Meier plotter was used to determine the relationship between the hub genes and survival time in BC patients, and evaluated the value of hub genes on BC prognosis.

## 2. Materials and methods

### 2.1 Microarray data

The gene expression dataset GSE66161^[19]^ and GSE70947^[20]^ were downloaded from Gene Expression Omnibus (GEO, http://www.ncbi.nlm.nih.gov/geo/) database. The samples of GSE66161 were breast tissues collected from 20 overweight or obese women who at increased risk of BC (greater than one in six lifetime risk). Researchers assessed the effect of IER intervention on gene expression in breast tissue during one menstrual cycle. The IER intervention consisted of two consecutive days of 65% ER (approximately 2700 kJ, 100 g carbohydrates and 50 g protein/day) and a Mediterranean type diet that met the estimated energy needs for the remaining five days of the week (range 7300 to 9500 kJ/day). The expression profiles of GSE70947 consisted of 105 breast adenocarcinomas and 105 matched adjacent normal breast tissue samples. The microarray platform used for GSE66161 was GPL570 ([HG-U133_Plus_2] Affymetrix Human Genome U133 Plus 2.0 Array) and GSE70947 was based on GPL13607 platform (Agilent-028004 SurePrint G3 Human GE 8x60K Microarray). All data were obtained and downloaded through the GEO database, so this study didn’t require approval from the ethics committee.

### 2.2 Data processing and DEGs screening

The raw microarray data of GSE66161 in CEL format was initially preprocessed into expression values through the Affy package in R software, and then a robust multiarray average (RMA) was formed by using background correction, normalization, and summarization. According to the annotation information on the platform, the probes were converted into the corresponding gene symbol. Probe set without corresponding gene symbols or genes with more than one probe set were removed or averaged, respectively. Analysing and characterizing the DEGs of samples before and after IER intervention with the threshold of |log Fold Change (log FC)| ≥ 0.584963 and P.value < 0.05 by using the Limma package in R software.

### 2.3 Function and pathway enrichment analysis of DEGs

Gene ontology (GO) annotations and Kyoto Encyclopaedia of Genes and Genomes (KEGG) pathways of DEGs were analysed by using Metascape database (http://metascape.org/) [21]. The conditions for screening significantly enriched GO terms and KEGG pathways were Min overlap = 3 and Min Enrichment = 1.5. P < 0.01 was considered statistically significant.

### 2.4 PPI network construction, module analysis and hub genes screening

We used STRING database to construct the PPI network, interaction with a combined score > 0.4 was selected and then Cytoscape software was used to visualize molecular interaction between proteins [22]. MCODE (Molecular Complex Detection), a plug-in of Cytoscape, can identify the most important module from PPI network. The selection criteria were as follows: degree cut-off = 2, node score cut-off = 0.2, Max depth = 100 and k-score = 2. The degree method was used to explore the hub genes in PPI network, and the screening condition was degrees ≥7.

### 2.5 Expression levels of hub genes in obesity

The expression levels of hub genes in obesity were verified by searching the Attie Lab Diabetes Database (http://diabetes.wisc.edu), an open database showing the expression levels of different genes in adipose tissue of both lean and obese BTBR mice (significance set at p < 0.05) [23].

### 2.6 Analysis of hub genes by GEPIA

GEPIA (http://gepia.cancer-pku.cn/) is a cancer big data analysis website consisted of The Cancer Genome Atlas (TCGA) and the Genotype-Tissue Expression (GTEx) portal [24]. In this study, GEPIA was used to investigate whether the hub genes were expressed differently in the breast tissues of 1085 BC patients between 291 normal people.

### 2.7 Predictive value of hub genes

To further investigate the predictive value of hub genes for postmenopausal BC risk, the performance of the signature of hub genes were evaluated by AUC of the ROC in GSE70947. According to the clinical features, postmenopausal women from GSE70947 were divided into group I (BMI<23.9 kg/m2) and group II (BMI≥24 kg/m2).

### 2.8 Survival analysis of hub genes

KM plotter is a survival analysis tool that using microarray data to evaluate the impact of certain genes on cancer prognosis [25]. To further discuss the impact of hub genes on the prognosis of BC patients, we identified overall survival (OS) and recurrence free survival (RFS) of hub genes among 7830 patients with BC by using KM plotter (http://kmplot.com/analysis/index.php?p=service&cancer=breast/).

## 3. Result

### 3.1 DEGs of samples before and after IER intervention

**Figure 1.**
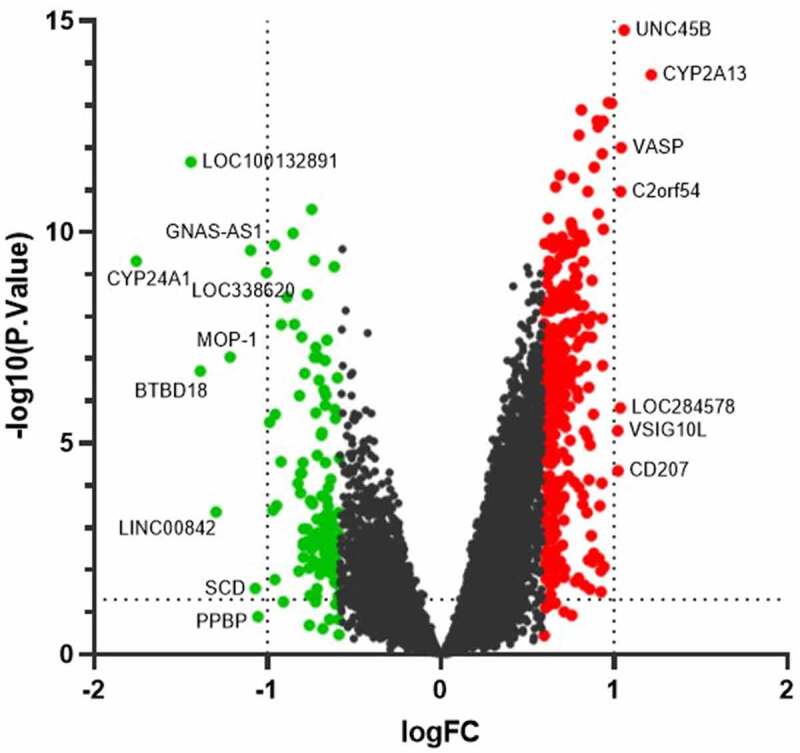
Volcano plot of DEGs (red points: up-regulated DEGs; green points: down-regulated DEGs). The differences were set as |log FC|≥ 0.584963. The significant DEGs with |log FC|≥ 1 were indicated with gene names.

**Figure 2.**
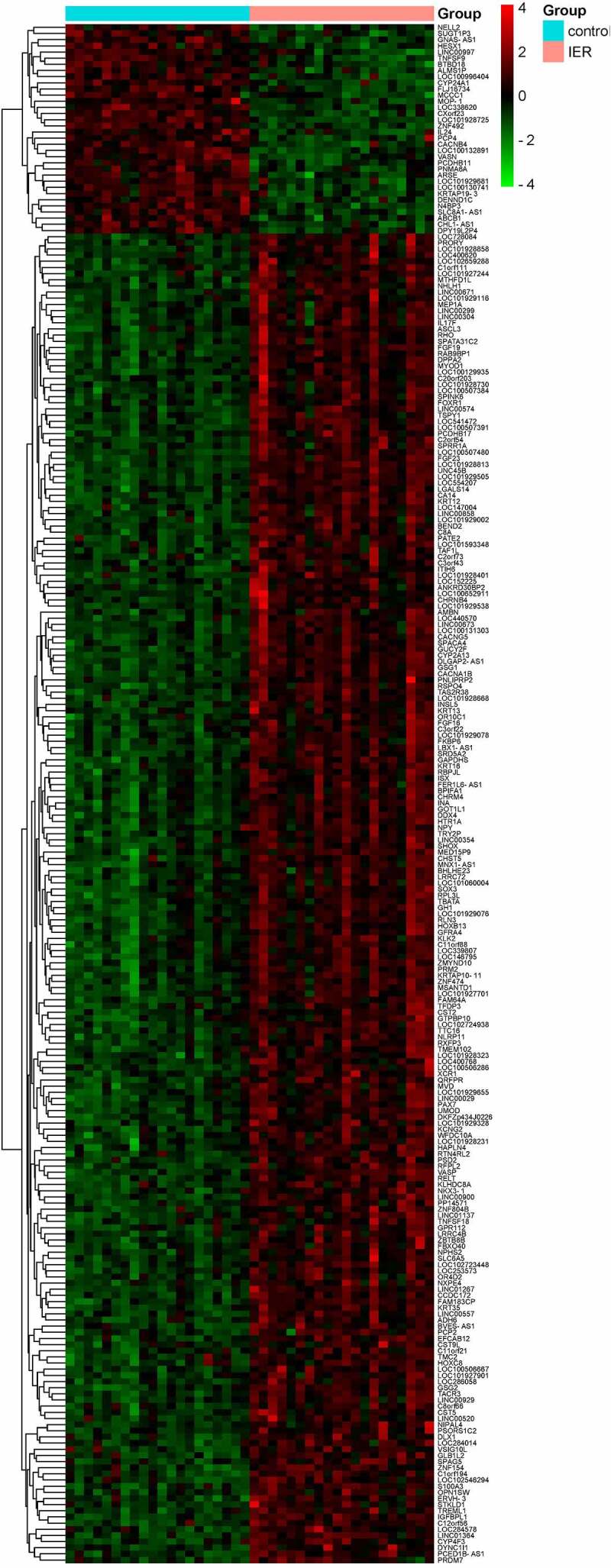
Heat map of DEGs (n = 250; blue bar: samples before IER intervention; pink bar: samples after IER intervention; red: up-regulated DEGs; green: down-regulated DEGs). Darker colour represented greater statistical significance.

We identified 331 up-regulated DEGs and 126 down-regulated DEGs in group after IER intervention compared to the group before IER intervention, and the results were displayed in the volcano map and heat map (Figures 1 and 2).

### 3.2 Function and pathway enrichment analysis of DEGs

We used Metascape database to perform the GO and KEGG pathway enrichment analyses of DEGs to further explore their functions and mechanisms in the reduction of BC risk. A total of 32 GO terms and 3 pathways of DEGs were derived from enrichment analysis, which including 14 BPs, 5 CCs, and 13 MFs (Figures 3 and 4). The result of GO enrichment indicated that there were main variations in BPs, including antimicrobial humoral response, detection of chemical stimulus involved in sensory perception of taste, fat-soluble vitamin catabolic process, and so on. The variations in CCs of DEGs were prominently enriched in the dense core granule, Golgi lumen, photoreceptor disc membrane, and so on. The variations in MFs were markedly enriched in peptide receptor activity, receptor ligand activity, signalling receptor activator activity, and so on. Analysis of KEGG pathway revealed that all DEGs were primarily enriched in Neuroactive ligand-receptor interaction, Cytokine-cytokine receptor interaction, and Phototransduction.

**Figure 3.**
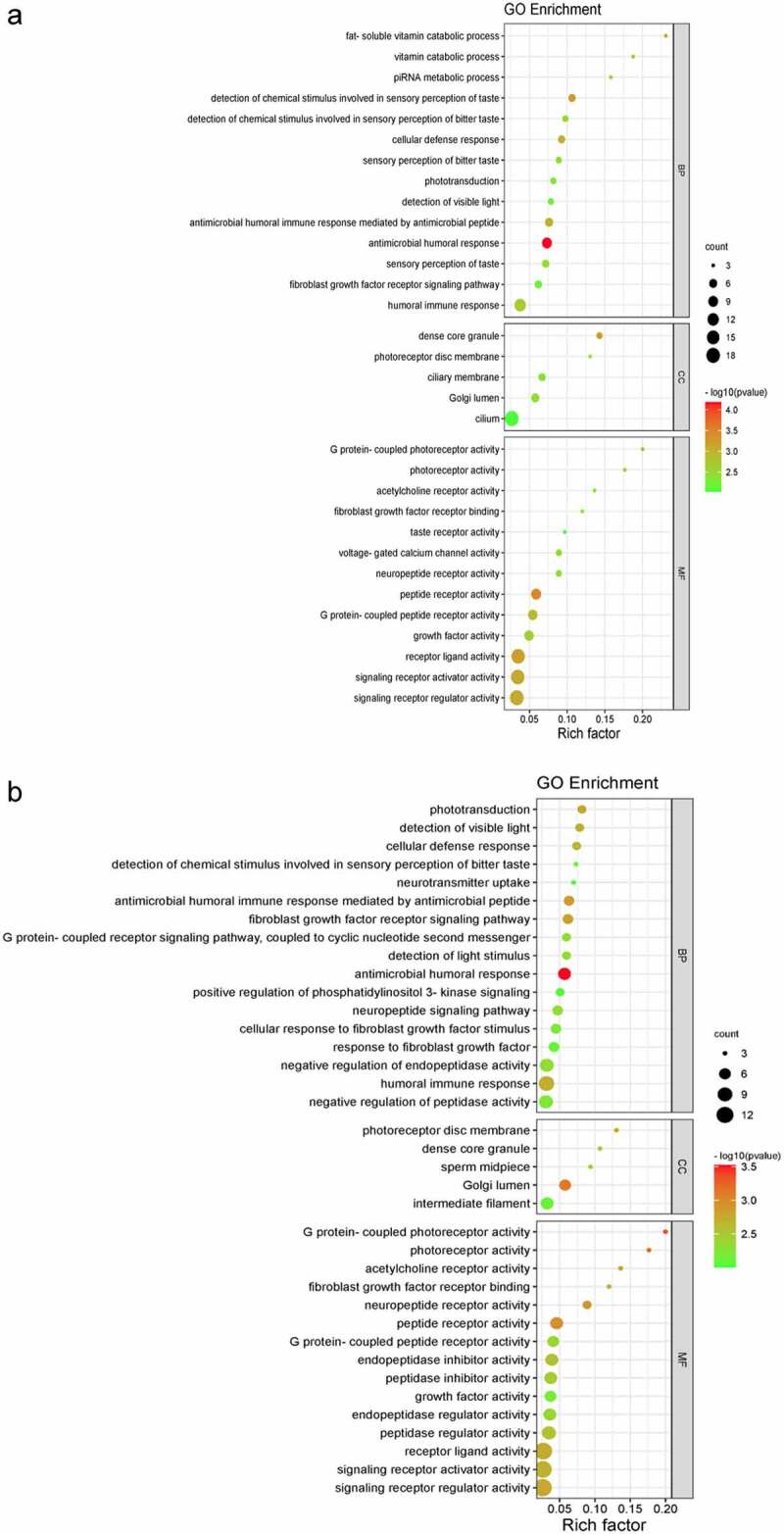
Bubble chart of GO Enrichment in three functional groups: biological processes (BP), cell composition (CC) and molecular function (MF). (a) all DEGs; (b) up-regulated DEGs; (c) down-regulated DEGs.

**Figure 3.**
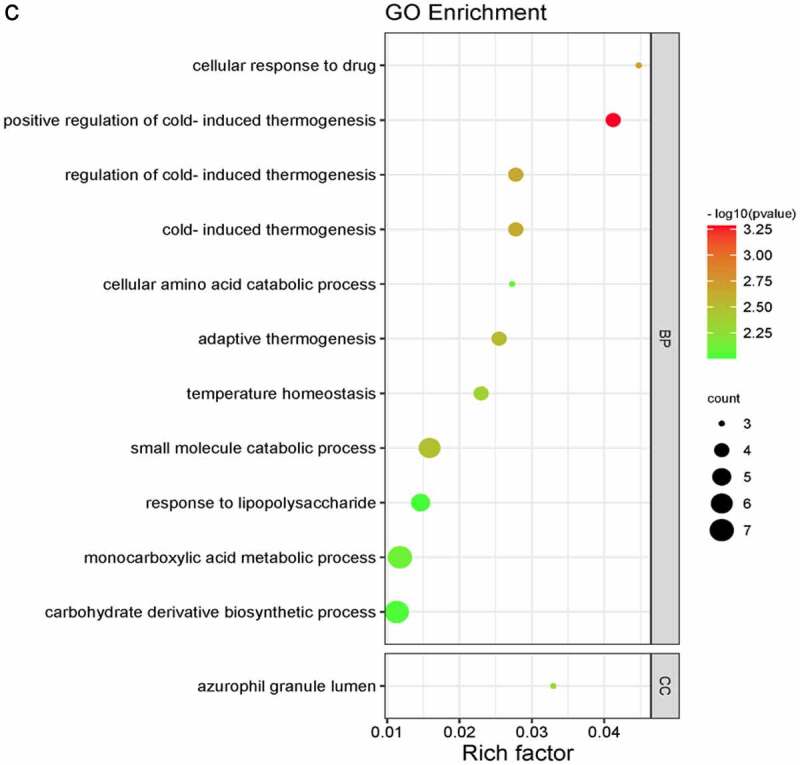
Bubble chart of GO Enrichment in three functional groups: biological processes (BP), cell composition (CC) and molecular function (MF). (a) all DEGs; (b) up-regulated DEGs; (c) down-regulated DEGs.

**Figure 4.**
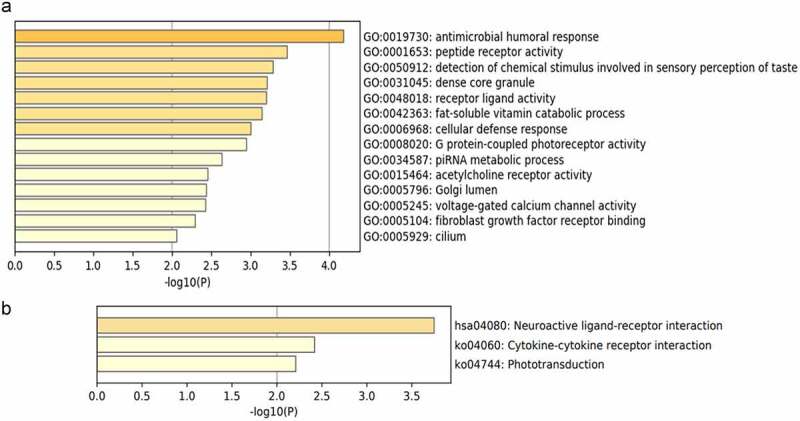
Heat maps of enriched GO pathway (a) and KEGG pathway (b), coloured by p-values.

### 3.3 PPI network construction, module analysis and hub genes screening

**Figure 5.**
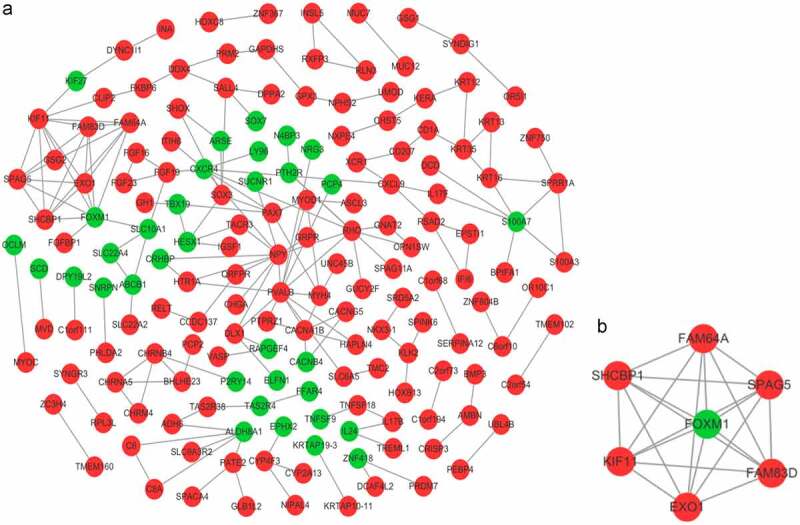
(a) Protein-protein interaction (PPI) network and (b) the most important module by using MCODE (red points: up-regulated DEGs; green points: down-regulated DEGs).

The PPI network of DEGs was constructed and visualized by the STRING database and Cytoscape, respectively. Results showed that there had 169 nodes and 187 edges in the PPI network (Figure 5a), and the most important module which identified by the MCODE was consisted of 6 up-regulated DEGs and 1 down-regulated DEG (MCODE score = 7.000, Figure 5b). The functional analyses of genes involved in this module were carried out by DAVID (http://david.ncifcrf.gov/), an online bioinformation database containing analytical tools, results showed that these 7 genes were primarily enriched in cytoplasm, protein binding, cell division and nucleus (Table 1), the KEGG pathway was not enriched.

**Table 1.** GO pathway enrichment analysis of 7 genes in the most important module

Pathway ID	Pathway description	Count in gene set	FDR
GO:0005737(CC)	cytoplasm	7	SPAG5, EXO1, FAM64A, KIF11, FOXM1, FAM83D, SHCBP1
GO:0005515(MF)	protein binding	6	SPAG5, EXO1, FAM64A, FOXM1, FAM83D, SHCBP1
GO:0051301(BP)	cell division	4	SPAG5, FAM64A, KIF11, FAM83D
GO:0005634(CC)	nucleus	4	SPAG5, EXO1, FAM64A, FOXM1
GO:0007067(BP)	mitotic nuclear division	3	FAM64A, KIF11, FAM83D
GO:0019901(MF)	protein kinase binding	3	KIF11, FOXM1, FAM83D
GO:0005654(CC)	nucleoplasm	3	SPAG5, EXO1, FOXM1

Abbreviations: GO, Gene Ontology; BP, biological processes; CC, cellular components; MF, molecular function.

**Figure 6.**
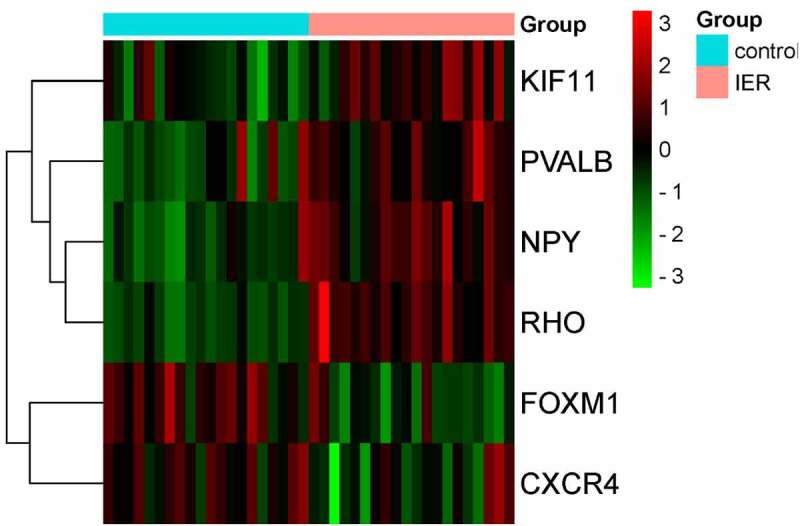
Heat map of 6 hub genes (blue bar: samples before IER intervention; pink bar: samples after IER intervention; green: down-regulated DEGs; red: up-regulated DEGs). Darker colour represented greater statistical significance.

A total of 6 genes (PVALB, NPY, KIF11, FOXM1, CXCR4, and RHO) were identified as hub genes with degrees ≥7 (Table 2). Among these genes, PVALB, NPY, KIF11 and RHO exhibited higher expression in the samples after IER intervention, while FOXM1 and CXCR4 were down-regulated. As showed in result (Figure 6), these hub genes could effectively distinguish the samples in the GSE66161 dataset before and after IER intervention, indicating that they may perform a key role in reducing the risk of BC after IER intervention.

**Table 2.** The names, full names and functional roles of 6 hub genes

Gene symbol	Full name	Function
PVALB	parvalbumin	encodes a high affinity calcium ion-binding protein
NPY	neuropeptide Y	encodes a neuropeptide that affects many physiological processes and is widely expressed in the central nervous system
KIF11	kinesin family member 11	encodes a motor protein that belongs to the kinesin-like protein family
FOXM1	forkhead box M1	encodes the protein which is a transcriptional activator involved in cell proliferation
CXCR4	C-X-C motif chemokine receptor 4	encodes a CXC chemokine receptor specific for stromal cell-derived factor-1
RHO	rhodopsin	encodes the protein which is in rod cells in the back of eye

### 3.4 Expression levels of hub genes in obesity

**Figure 7.**
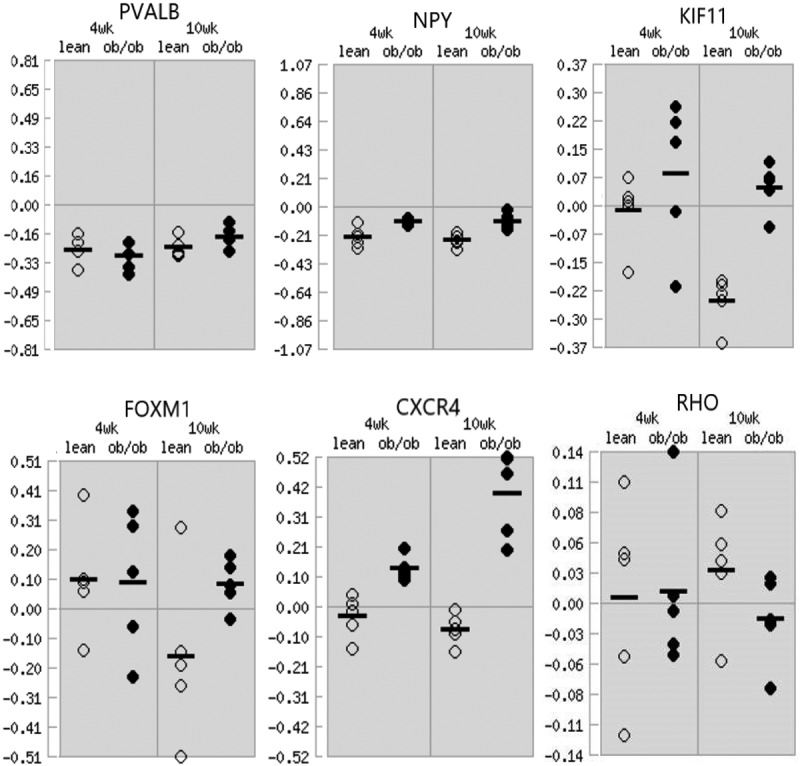
Expression levels of hub genes in the adipose of lean/obese mice. The y-axis label represents ratio.

The expression levels of hub genes in obesity were verified by applying Attie Lab Diabetes database. The results showed that the expression levels of PVALB, NPY, KIF11, FOXM1 and CXCR4 were up-regulated in adipose of obese mice at 10 weeks compared with the lean group (Figure 7).

### 3.5 Analysis of hub genes by GEPIA

**Figure 8.**
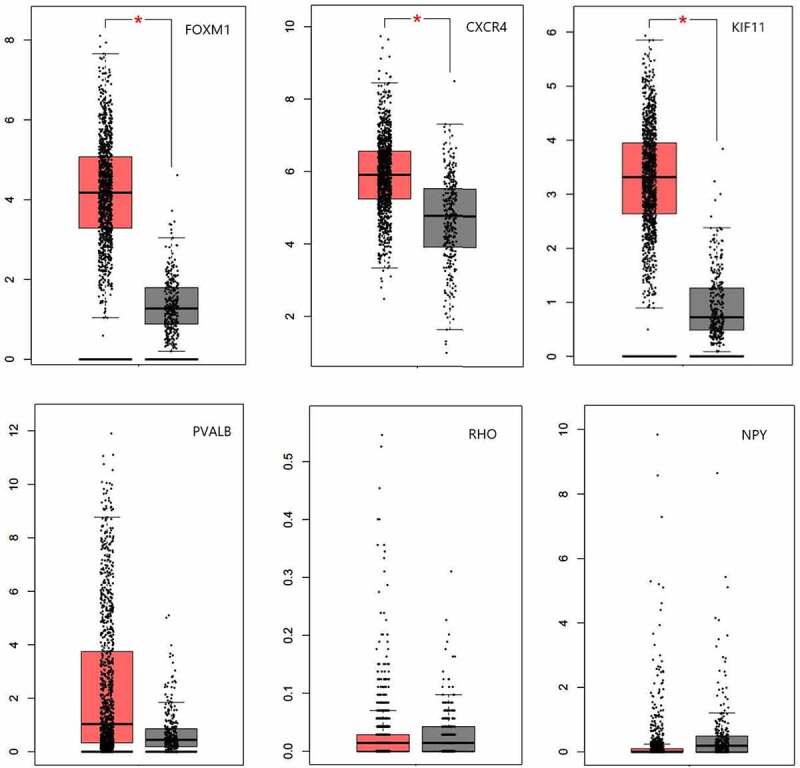
Six hub genes in BC patients compared to normal people. Red means breast cancer tissues and grey means normal breast tissues (|log FC|>1; P < 0.01).

We used GEPIA to demonstrate the expression level of hub genes in normal breast tissues and BC tissues. The results revealed that FOXM1, CXCR4 and KIFF11 were highly expressed in BC tissues compared with normal breast tissues, and the difference was statistically significant (Figure 8).

### 3.6 Predictive value of hub genes

**Figure 9.**
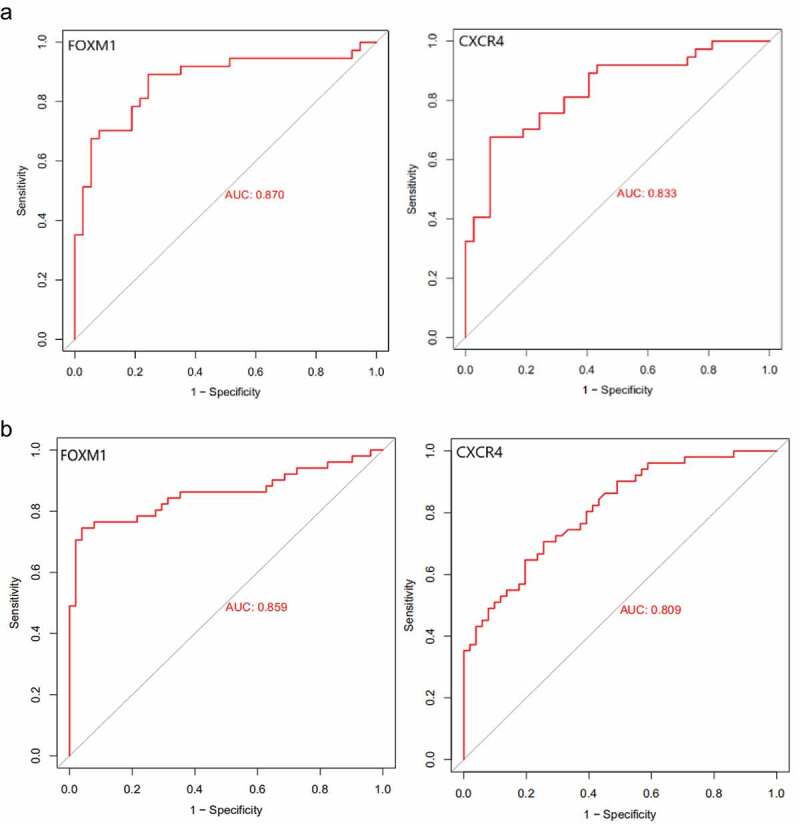
Receiver operating characteristic (ROC) curves of FOXM1 and CXCR4 in the GSE70947 datasets. (a) group I (BMI<23.9 kg/m2); (b) group II (BMI≥24.0 kg/m2).

Among hub genes which had high expression in BC tissues, FOXM1 and CXCR4 exhibited the lower expression in the samples after IER intervention, while KIF11 was up-regulated. We speculated that FOXM1 and CXCR4 may be the most important key genes for postmenopausal BC risk reduction after IER intervention. ROC curve was used to further validate the predictive value of FOXM1 and CXCR4 in postmenopausal BC. Results indicated that FOXM1 and CXCR4 exhibited excellent diagnostic efficiency for BC in postmenopausal women (Figure 9). AUC of FOXM1 and CXCR4 in group I (BMI<23.9 kg/m2) were 0.870 and 0.833, respectively. AUC of FOXM1 and CXCR4 in group II (BMI≥24.0 kg/m2) was 0.859 and 0.809, respectively.

### 3.7 Survival analysis of FOXM1 and CXCR4

**Figure 10.**
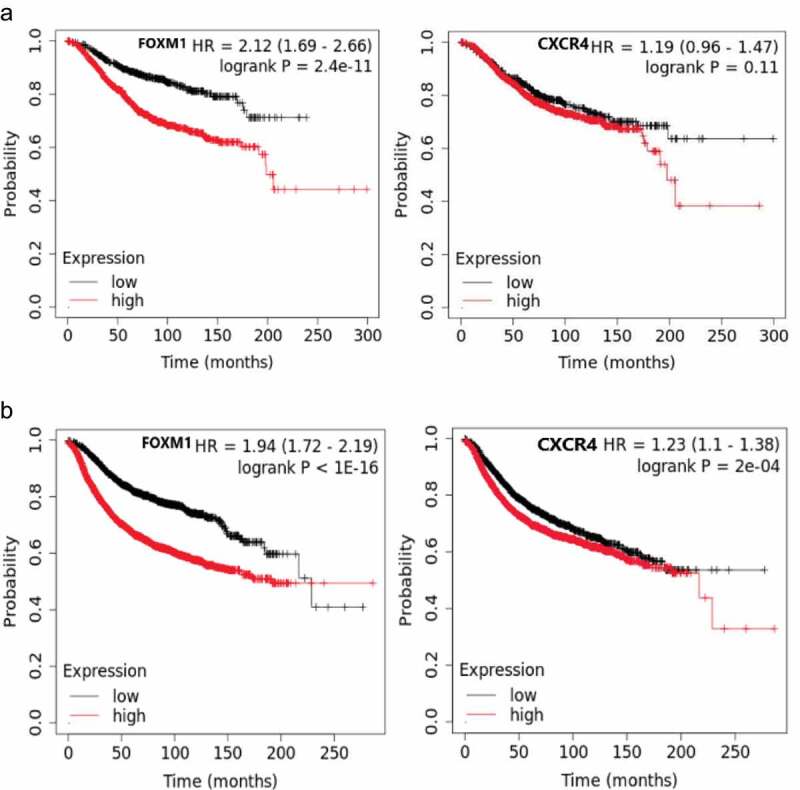
Overall survival (a) and recurrence free survival (b) of FOXM1 ad CXCR4 (P < 0.05).

To further identify whether FOXM1 and CXCR4 would affect the survival time of BC patients, we identified the relationship between mRNA level and the clinical prognosis of BC. The results of survival analysis had showed that high expression of FOXM1 resulted the poor OS and RFS in BC patients. As for CXCR4, high expression of CXCR4 in BC promoted the recurrence of BC, but didn’t have a significant impact on OS (Figure 10).

## 4. Discussion

Obesity, as a known risk factor, not only increases the risk of postmenopausal BC, but also worsens the prognosis for patients with BC [26,27]. A large number of observational studies and large randomized diet trials have confirmed that weight loss and ER could reduce the occurrence of postmenopausal BC in women [5,12,28,29]. Although preclinical studies support the possibility that ER may reduce the risk of cancer by stimulating anti-cancer immune pathways [30], the lacking of strong enough evidences to draw definitive conclusions still requires more studies to investigate the mechanisms between ER and BC.

Based on the GEO dataset (GSE66161), this study used integrated bioinformatics analysis to explore the changes of key gene expression in breast tissues after IER intervention to understand the potential pathways of IER in reducing the risk of postmenopausal BC. We identified 331 up-regulated DEGs and 126 down-regulated DEGs for subsequent analysis, and the enrichment analysis showed that these genes were prominently enriched in BP. Up-regulated DEGs were related to antimicrobial humoral response, photoreceptor activity, peptide receptor activity, acetylcholine receptor activity and fibroblast growth factor receptor (FGFR) signalling pathway. And the down-regulated genes were closely related to positive regulation of small molecule catabolic process, monocarboxylic acid metabolic process, cellular amino acid catabolic process and carbohydrate derivative biosynthetic process, which demonstrated that these DEGs after IER intervention were significantly enriched in the energy metabolism. Metabolic alterations are the hallmark of cancer, and reprogramming of energy metabolism has long been considered as a common phenomenon in tumours [31]. Some of the most significant biometric changes in tumour cells include elevation of glycolysis, upregulation of amino acid, lipid metabolism and molecule biosynthesis [32]. The result of enrichment analysis in our study suggested that some genes related to metabolic synthesis and energy production pathways were down-regulated in breast tissue following IER intervention. This is consistent with the results of Meynet O and Fabian CJ [33,34], that the pathways related to anabolism are down-regulated while pathways related to catabolism are up-regulated after ER. Cancer is a disorder of cell growth and proliferation, besides high amounts of energy, the cellular building blocks are also required necessarily, including nucleic acids, proteins, and lipids [35]. The most important module identified from PPI network was also mainly enriched in cytoplasm, protein binding, cell division and nucleus, which was consistent with the biological characteristics of the abnormally rapid proliferation of BC cells.

The degree method was used to identify the hub genes in PPI network, and a total of 6 genes (PVALB, NPY, KIF11, FOXM1, CXCR4, and RHO) were identified as hub genes with degrees ≥7. Obesity is a significant risk factor and negative prognostic factor for BC, and the adipocyte-derived adipokine links obesity and BC by playing a key role in energy metabolism, food intake, and endocrine systems, among others [36]. In order to confirm the association between hub genes and obesity, we obtained the expression levels of hub genes in adipose from Attie Lab. Results showed that the expression levels of PVALB, NPY, KIF11, FOXM1 and CXCR4 in adipose tissue of obese mice were higher than those of lean mice, which meant that hub genes after IER intervention were strongly correlated with obesity. RHO was not up-regulated in obesity may be due to the fact that the protein encoded by RHO mainly act on vision in low-light conditions and didn’t play a role in obesity-related pathways. FOXM1, CXCR4 and KIFF11 were overexpressed in BC tissues compared to normal breast tissues, while PVALB, NPY and RHO expression between the two groups didn’t have significant differences. However, considering the down-regulation of FOXM1 and CXCR4 expression level in breast tissues after IER intervention, we speculated that FOXM1 and CXCR4 might be the most important key genes in reducing the risk of postmenopausal BC after IER intervention. FOXM1 plays a key role in proliferation and cell cycle progression through transcriptional activation of a G2/M-specific gene network [37], and increased FOXM1 gene expression and its transcriptional signature have been detected in many cancer types [38]. The chemokine receptor CXCR4 in adipose tissue is expressed on adipocytes [39] and is highly expressed in various types of cancers by inducing chemotactic and invasive responses and mediating actin polymerization and pseudopodia formation [40]. KIF11 is a molecular motor protein that plays essential role in mitosis [41]. In our study, energy production pathways were down-regulated in breast tissue after IER, while energy consumption pathways were up-regulated. The expression level of KIF11 in breast tissue after IER was up-regulated may be related to the up-regulation of cell mitosis due to its high energy consumption. The ROC curve revealed that the expression level of FOXM1 and CXCR4 in postmenopausal women from GSE70947 could predict BC sensitively and specifically, which meant that FOXM1 and CXCR4 also had the predictive value for postmenopausal BC.

Overweight or obese is associated both with a higher risk of worse BC outcome for women of all ages [42]. ER has a wide impact on several cellular processes including adipokine expression [43,44], at the same time, our study has found that metabolic synthesis pathways were down-regulated in breast tissue after IER. We speculated that IER would improve BC outcome by inhibiting adipocyte-derived adipokine production and metabolic synthesis pathways. FOXM1 and CXCR4 maybe also the potential targets for improving the prognosis of BC. As showed in survival analysis, high FOXM1 expression was associated with poor OS and RFS of BC. Although the molecular mechanisms of FOXM1 on improving BC progression are poorly understood, there is the strong possibility that FOXM1 will be a promising therapeutic target for cancer treatment. Patients with high expression level of CXCR4 had worse RFS of BC, but didn’t have a significant impact on OS, which was consistent with a previous study reported by Guo K, et al [45]. However, one meta-analyses from Wang F, et al [46] demonstrated that high CXCR4 mRNA expression in BC patients often resulted in shorter overall survival time. There are some influence factors disturbing OS, such as non-cancer-related mortality. But anyway, low CXCR4 expression is associated with better prognosis of BC, larger samples and higher-quality studies are needed to reinforce the credibility of this argument.

FOXM1 and CXCR4, as the most important key genes, were overexpressed in obesity and BC, and its overexpression was associated with high occurrence and worse outcome of BC. This study provided a partial theoretical basis for the further study of IER inducing changes of gene expression in breast tissues, and provided a new intervention method and potential targets for the clinical prevention and treatment of BC. However, the limitations of our study also should be recognized. First of all, only 20 samples were included in GSE66161, and the study didn’t include other ethnic groups. The above findings are needed to be validated in independent larger datasets including in vivo/in vitro laboratory performance. Secondly, there was not subgroup analysis according to the BC molecular subtypes and menopause status. More studies based on the larger sample size are required to reveal the preventive and prognostic value of FOXM1 and CXCR4 in different BC molecular subtypes and menopause status.

## 5. Conclusion

In summary, it had been found that the expression of multiple genes in breast tissues was altered after IER intervention in overweight or obese women. We concluded that IER induced a great change in gene enrichment pathways from a state of energy production to a state of energy inhibition in breast tissue, with reduction in metabolic function, which represented the crucial mechanisms after IER intervention. In this study, we identified six hub genes in samples after IER intervention, uncovered the possible pathways, and analysed the important underlying mechanisms that IER intervention maybe involved in. FOXM1 and CXCR4 were down-regulated after IER intervention, which meant that FOXM1 and CXCR4 might be the most important key genes in reducing the risk of postmenopausal BC after IER intervention. Meanwhile, the overexpression of FOXM1 and CXCR4 was associated with poor patient outcome of BC. We speculated that IER intervention would improve BC outcome and FOXM1 and CXCR4 may also be the potential targets for improving prognosis of BC. Our results contributed to a better understanding of the specific mechanisms in protective effects of IER on BC and provided a new approach to improve the prognosis of BC, which might provide partial guidance for the subsequent development of more effective treatments and prevention strategies.

## Data Availability

The data used in this study can be found and downloaded from the GEO database (https://www.ncbi.nlm.nih. gov/geo/), reference number 19, 20.
